# Comparing the Relaxing Effects of Different Virtual Reality Environments in the Intensive Care Unit: Observational Study

**DOI:** 10.2196/15579

**Published:** 2019-11-15

**Authors:** Stephan M Gerber, Marie-Madlen Jeitziner, Simon D Sänger, Samuel E J Knobel, Laura Marchal-Crespo, René M Müri, Joerg C Schefold, Stephan M Jakob, Tobias Nef

**Affiliations:** 1 Gerontechnology & Rehabilitation Group University of Bern Bern Switzerland; 2 Department of Intensive Care Medicine, University Hospital Bern (Inselspital), University of Bern Bern Switzerland; 3 Department of Neurology, University Neurorehabilitation, University Hospital Bern (Inselspital), University of Bern Bern Switzerland; 4 ARTORG Center for Biomedical Engineering Research University of Bern Bern Switzerland

**Keywords:** virtual reality, critical illness, intensive care unit, neurocognitive late effects, nature, urban, stimulation

## Abstract

**Background:**

After a prolonged intensive care unit (ICU) stay, approximately 50%-75% of all critically ill patients suffer from neurocognitive late effects and a reduction of health-related quality of life. It is assumed that the noisy and stressful ICU environment leads to sensory overload and deprivation and potentially to long-term cognitive impairment.

**Objective:**

In this study, we investigated three different virtual reality environments and their potentially restorative and relaxing effects for reducing sensory overload and deprivation in the ICU.

**Methods:**

A total of 45 healthy subjects were exposed to three different environments, each 10 minutes in length (dynamic, virtual, natural, and urban environments presented inside the head-mounted display, and a neutral video on an ICU TV screen). During the study, data was collected by validated questionnaires (ie, restoration and sickness) and sensors to record physiological parameters (240 hertz).

**Results:**

The results showed that the natural environment had the highest positive and restorative effect on the physiological and psychological state of healthy subjects, followed by the urban environment and the ICU TV screen.

**Conclusions:**

Overall, virtual reality stimulation with head-mounted display using a dynamic, virtual and natural environment has the potential, if directly used in the ICU, to reduce sensory overload and deprivation in critically ill patients and thus to prevent neurocognitive late effects.

## Introduction

Approximately 50%-75% of critically ill patients experiencing a prolonged intensive care unit (ICU) stay will suffer from neurocognitive late effects [1,2]. Neurocognitive late effects (eg, deficits in learning and memory, slowed information processing, attention, concentration, and executive function) of critically ill patients have been associated with a reduced ability to cope with everyday activities and a significant reduction in quality of life after discharge [2-5]. In addition to the medical condition of critically ill patients itself (eg, organ failure, delirium, and muscle weakness and atrophy), the stressful environment is also an important contributor to neurocognitive late effects [6,7]. ICU patients are exposed to high noise levels, artificial light, and isolation from their common environment, which leads to sensory overload and deprivation and potentially to long-term cognitive impairment [7-9]. There is evidence that the early introduction of an intervention in the ICU has the greatest impact on long-term outcome [1,10]. Current clinical practice in the ICU to reduce functional impairment includes early mobilization and physiotherapy, but there is less consensus about how to reduce or prevent neurocognitive late effects [11].

We propose using virtual reality (VR) technology with head-mounted display to comfort and reduce the stress of ICU patients. A state-of-the art, head-mounted display for the presentation of stereoscopic images and three-dimensional sounds (developed for the gaming industry) provides sensors for measuring head movements, which allows the user to look around in the virtual environment. Thus, wearing a head-mounted display enables patients to experience virtual environments with their visual and auditory senses while being highly immersed [12]. Since vision and hearing are completely controlled by the head-mounted display, patients do not notice anything from their surroundings, thus helping them avoid the noisy and stressful ICU environment. The virtual environment presented inside a head-mounted display is a computer-generated, visual, and auditory replication of a real (eg, a forest or city) or imaginary (eg, a futuristic city) environment. Virtual environments must be designed carefully to avoid negative side effects (eg, cybersickness, oculomotor problems) [13,14]. This leads us to the question of what virtual environment would comfort ICU patients the most.

In a previous study, we showed that VR exposure to a virtual environment that replicates beautiful nature scenes is feasible and potentially beneficial for critically ill patients. The stimulation had a strong relaxing effect without any adverse effects. Participants reported that the VR exposure was calming and that they were immersed in the virtual environment [15]. Our head-mounted display complied with hygiene requirements in the ICU, and patients could use the head-mounted displays while lying in bed. Based on these preliminary results, we believe that head-mounted displays can be used as an early rehabilitative intervention to provide cognitive stimulation with the goal of reducing neurocognitive late effects.

In the present study, we compare the relaxing effects of a virtual nature environment with a virtual urban environment, in combination with noise-cancelling headphones. As a control condition, we selected watching a movie about intuition on a TV screen with loudspeakers. This choice was made because in the ICU patients are often invited to watch TV. We hypothesize that the relaxing effects of the natural environment will be greater than the urban environment, and both greater than the control condition. This expectation is based on two theories that propose that restorative environments restore neurocognitive late effects (ie, attention), physiological and emotional functions, and have protective effects against environmental stressors. First, the stress recovery theory states that restorative environments enable us to relax and recover from stress and mental fatigue, as seen in markers of physiological stress [16]. Second, the attention restoration theory claims that restorative environments enable us to restore depleted resources of attention since effortless attention (ie, cognition) is forced [17-19]. Therefore, an effective restorative environment must be rich, harmonious, soft, fascinating, and give the feeling of being away [19]. Natural environments are especially suitable for restoring depleted attention resources in a structured and effortless way [20,21]. Additionally, restoration when viewing static nature images is faster and more complete compared to urban images, and there is evidence that being present in the restorative environment has comparable restorative effects to virtual environments presented in a head-mounted display [16,19,22].

In this study, to measure the restorative effect and thus the reaction to the stimulation, we used questionnaires about perceived restoration and sickness (ie, the Perceived Restoration Scale [PRS] and the Simulator Sickness Questionnaire [SSQ]) in combination with standard medical physiological measures (eg, heart rate, respiration rate, blood pressure) to measure implications on the parasympathetic nervous system.

## Methods

### Participant Recruitment and Demographics

The study was conducted following the current version of the Declaration of Helsinki. All subjects participating in the study were recruited through the University of Bern, and the study was approved by the Ethics Committee of the Canton of Bern, Switzerland (KEK-Nr. 2017-02195). All subjects signed written informed consent before inclusion. Furthermore, specific consent was obtained to publish identifying information and images in an online open-access publication. The main exclusion criteria were auditory and visual impairments and being below the age of 18.

### Neurocognitive Stimulation

The study was conducted in a one-bed cubicle in the ICU of the University Hospital Bern. To stimulate the subjects, either a head-mounted display (HTC Vive, High Tech Computer Corporation, Taoyuan, Taiwan) or a classical ICU TV screen (MediTec TV, Bewatec, Telgte, Germany) was used. The head-mounted-display had a resolution of 1080 × 1200 pixels per eye, a field of view of 110 degrees, and a refresh rate of 90 hertz (Hz), whereas the ICU TV screen had an 11.5-inch display and an aspect ratio of 4:3.

**Figure 1:**
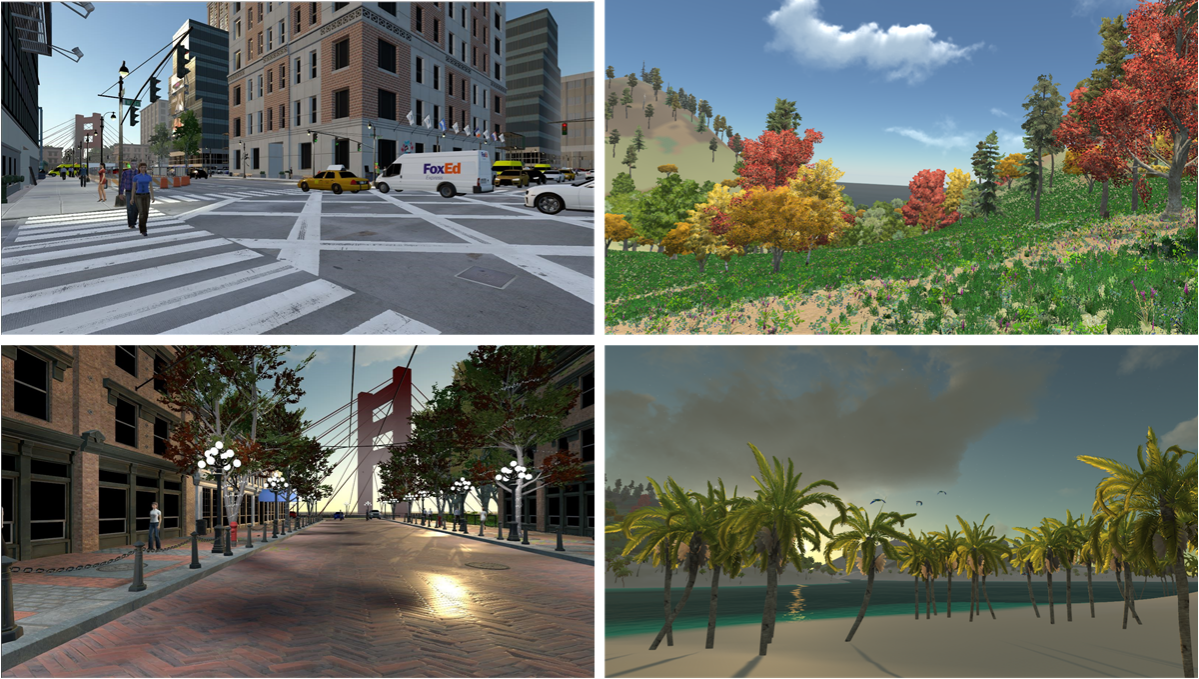
Left: VR urban environment; right: VR nature environment. VR: virtual reality.

Subjects were stimulated two times with the head-mounted display (ie, virtual nature and urban VR stimulation, Figure 1) and once with the gold standard (control condition), a classical ICU TV screen (movie), with each interaction lasting 10 minutes. The nature VR stimulation consisted of a large island surrounded by water, with green hill areas, beaches, forests, plantations, and several animals such as elephants, giraffes, dolphins, birds, and butterflies. The designed urban VR stimulation consisted of a busy downtown, followed by a more relaxed old town. Pedestrians, cars, birds, aircrafts, and a construction site were among the many objects that could be found in the environment. In both cases, subjects were walking on a predefined path. In the control condition, a neutral documentary movie about intuition was presented (ie, combination of nature and urban scenes).

Physiological parameters (ie, noninvasive arterial blood pressure, heart rate, and respiratory rate) were monitored by the in-house monitor system of the University Hospital (Carescape Monitor B650, GE Healthcare, Little Chalfont, United Kingdom). Respiration and heartrate were measured at a frequency of 240 Hz, whereas blood pressure was measured every second minute. To simulate a real ICU scenario, the physiological monitor system constantly produced alarm sounds (eg, simulated arrhythmia). In addition, a noise meter was set up next to the subject to monitor and guarantee the overall noise level in the room was above 50 decibels. The bed was tilted up so that the subjects were in an upright position. The system was approved by the University Hospital’s medical-technical department, except for use in critically ill patients and subjects (ie, hygiene and medical eligibility approved). To evaluate the perceived restorativeness of each stimulation, the PRS-11[23] (measured by a seven-point scale from not at all to completely) and for discomfort the SSQ [24] (measured by a four-point scale, from none to severe) were used. Both score scales were normalized on a score scale between zero and one.

### Study Design and Procedure

**Figure 2:**
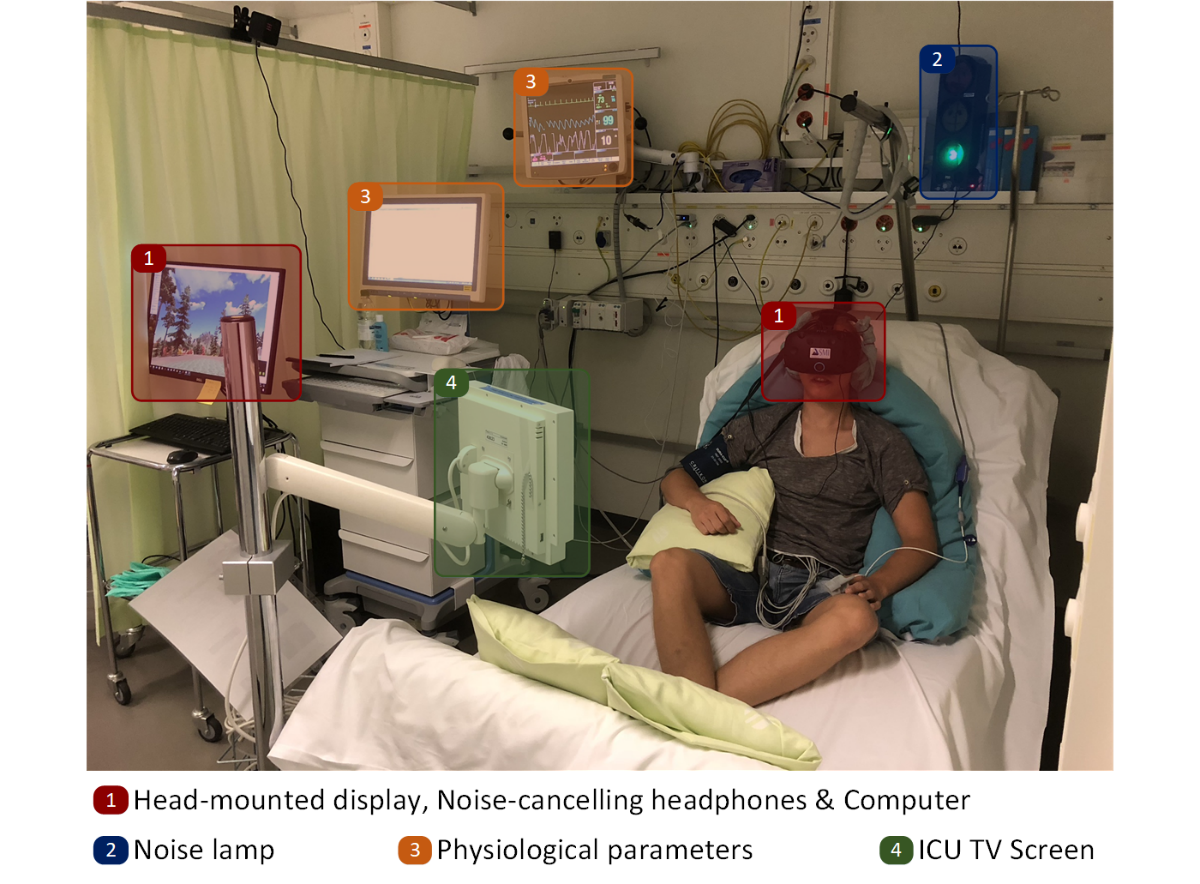
Subject during the stimulation in a VR environment, including the whole setup. ICU: intensive care unit; VR: virtual reality.

To account for the influence of the order of the stimulations, the subjects were randomly assigned in a pseudo cross-validation to one of the three groups with different stimulation sequences. First, subjects were instructed and prepared (ie, lying on the bed, Figure 2) for the experiment, followed by a recovery phase of 10 minutes to stabilize physiological parameters at subjects’ baseline. Next, subjects were assigned randomly to one of the three groups and stimulated with the three environments with a break of approximately 10 minutes in between. After each stimulation, they were asked to fill out a questionnaire of the perceived restorativeness and discomfort. Before the stimulation, subjects filled out a questionnaire about demographics. All instructions were given prior to the stimulation, and participants could move their head to explore the environment while seated. For each participant, the experiment lasted approximately 80 minutes.

### Statistical Analysis

To analyze the calming and relaxing effect (ie, negative time effect is defined as the reduction of physiological parameters during the course of the stimulation) of an environment, the correlation between time and the physiological measurement was calculated and tested on their significance using a one-tailed *t* test against zero. To analyze the differences in questionnaire ratings between the stimulations, one-way analysis of variance (ANOVA) was used, followed by a *post hoc* analysis using paired *t* tests and Bonferroni correction for *P* value adjustment. The analysis of the physiological parameters and the questionnaires was conducted by using R for statistics (The R Foundation, Vienna, Austria) and MATLAB 2018b (MathWorks, Natick, USA).

### Website

A video of the two VR stimulations and the movie about intuition can be found on our website [25].

### Data and Code Availability

All relevant codes and data supporting the findings are presented in this paper. Further code and data of the study are available upon request.

## Results

### Perceived Restoration

A total of 45 (22 male and 23 female) adults between the ages of 22 and 87 (mean 59; SD 16) participated in the study. Overall, 29 participants grew up in the countryside and 16 in an urban environment, and they were all in good health. Analysis using ANOVA showed a significant difference between the three stimulation environments (F_2_=15.86; *P*<.001). The nature VR stimulation had the highest perceived restoration (mean 0.773; SD 0.142) and was closest to the maximum of the score scale (with none=0 and high=1), followed by urban VR (mean 0.65715; SD 0.187) and ICU TV stimulation (mean 0.5854; SD 0.136). A *post hoc* analysis showed a significant difference between nature VR and both urban VR (*P*=.002) and ICU TV (*P*<.001), but not between urban VR and ICU TV stimulation (*P*=.12).

**Figure 3:**
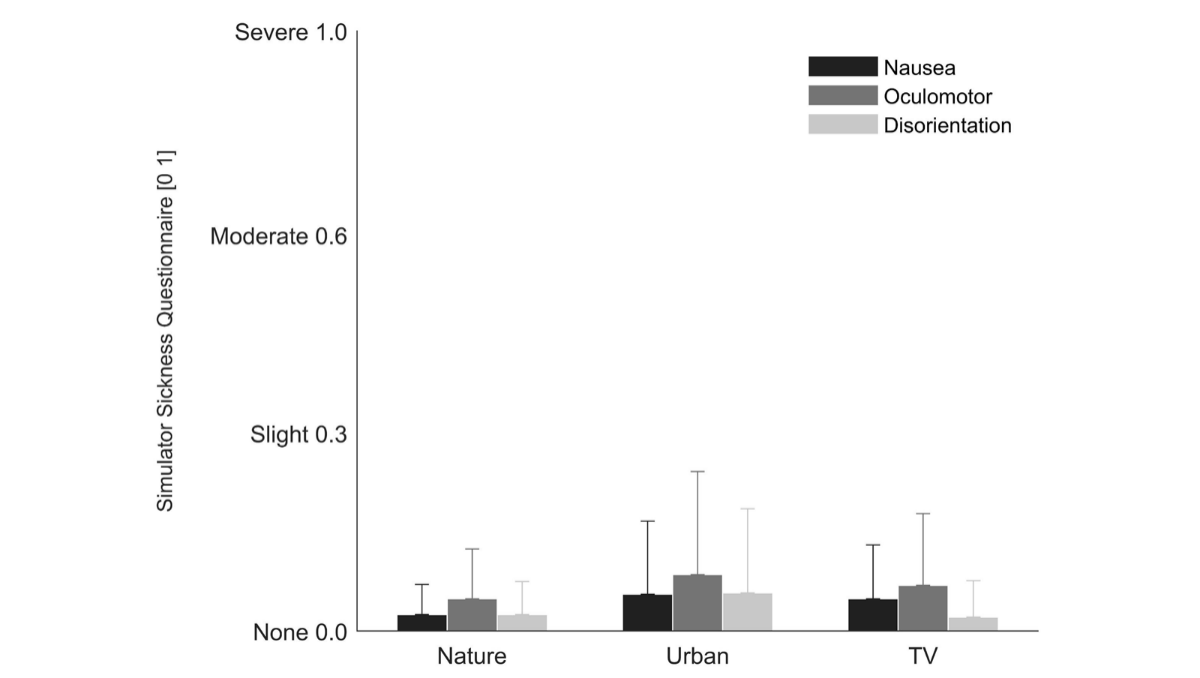
The results of the Simulator Sickness Questionnaire for each of the three environments.

In all three environments, nausea, oculomotor problems, and disorientation were close to the minimum of the score scale (Figure 3). The only significant difference in discomfort between the environments was found in disorientation (F_2_=3.67; *P=*.03).

### Physiological Measurements

**Figure 4:**
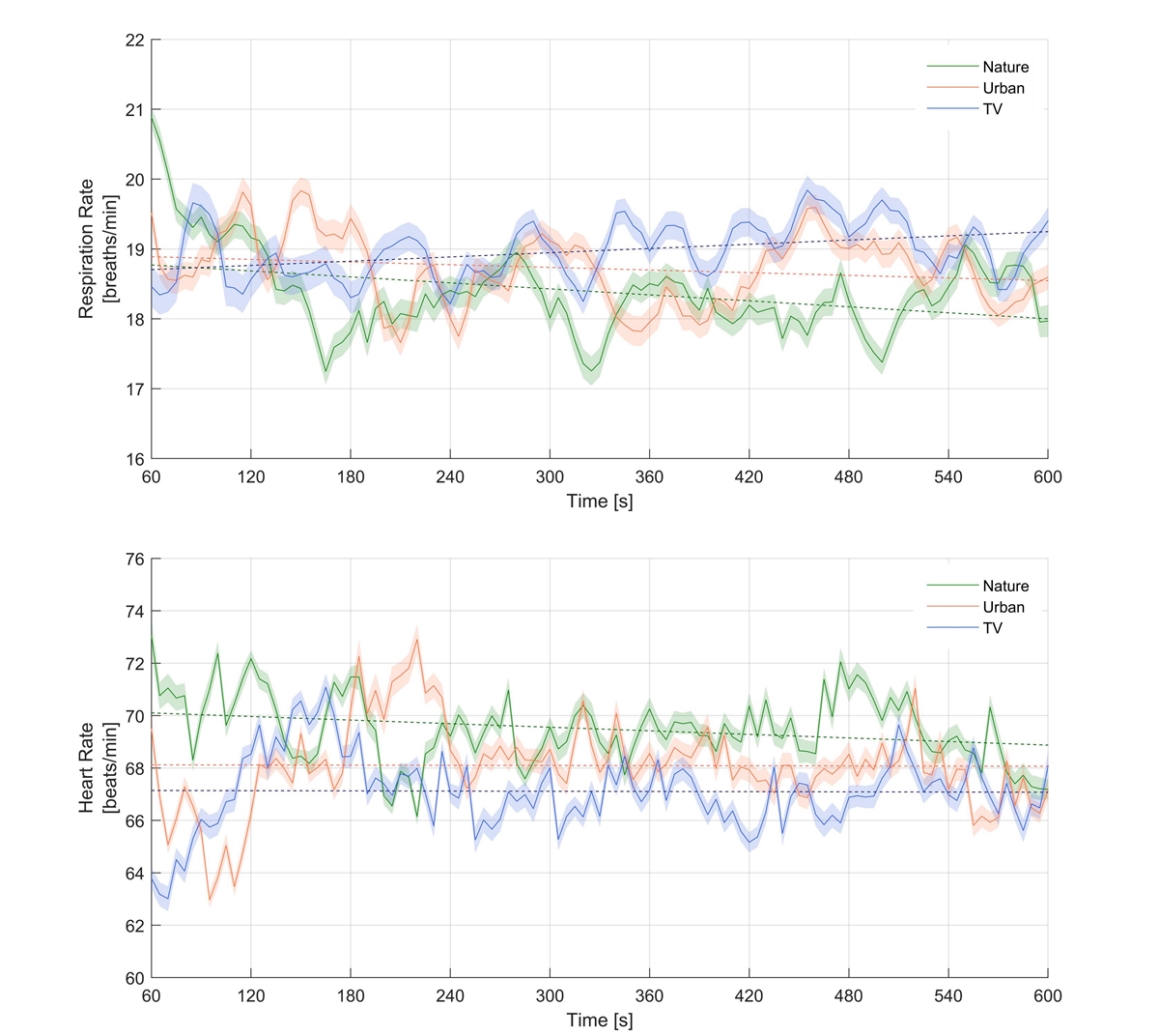
Heart- and respiration rate of the three stimulation environments. In all cases, the standard deviation is shaded with the same color.

At the beginning of the recovery phase, respiration rate was an average of 19.35 Breaths/minute (SD 5.08), heart rate was an average of 69.88 Beats/minute (SD 11.03), and the mean blood pressure was 94.3 mmHg (SD 11.81). As shown in Figure 4, nature and urban VR stimulation were associated with a reduction in respiration rate, whereas during ICU TV stimulation respiration rate increased. The negative correlation of respiration rate and time (negative time effect) was highest and only significant in nature VR stimulation (95% CI –Inf to –0.024; *t*_42_=−2.13; *P*=.02), but neither in urban VR stimulation (95% CI –Inf to 0.052; *t*_40_=−0.69; *P*=.25) nor in ICU TV stimulation (95% CI –Inf to 0.132; *t*_41_=1.31; *P*=.90). With respect to heart rate, only nature VR decreased during the course of the stimulation, whereas in urban VR and ICU TV stimulation heart rate remained more or less constant. However, there was a significant negative time effect in heart rate in nature VR stimulation (95% CI –Inf to –0.177, *t*_2_=−7.53; *P*<.001), in urban VR stimulation (95% CI –Inf to 0.161; *t*_40_=−7.35; *P*<.001) and in ICU TV stimulation (95% CI –Inf to 0.141; *t*_38_=1.31; *P*<.001). Furthermore, the mean blood pressure did not increase or decrease during any of the three stimulations.

## Discussion

### Primary Results

In this study, we investigated the subjective restorative and relaxing effect of three different stimulation methods (dynamic, virtual, nature and urban environments presented inside a head-mounted display and a neutral video on an ICU TV screen) in healthy subjects. In line with our hypothesis, there was a significantly higher restorative and relaxing effect in the virtual nature environment compared to the virtual urban environment and the ICU TV screen. Additionally, in all cases, the three different methods evoked none to minor negative reactions.

### Perceived Restoration and Comfort

The main finding in line with the attention restoration theory was that the highest perceived restorative effect was found in virtual nature stimulation, followed by the virtual urban stimulation and the movie presented on the ICU TV screen [19,26]. Furthermore, the three different stimulation methods showed none to minor negative side effects, like nausea, oculomotor problems, or disorientation. The slightly higher discomfort in the virtual urban environment was possibly due to unexpected 90 degree turns (ie, when walking around a building corner), whereas in the virtual nature environment no turns were present.

### Physiological Measurements

The second main finding was that the nature VR stimulation with head-mounted display revealed the highest and only significant reduction in respiration rate. In case of heart rate, only the nature VR stimulation showed a relaxing effect, whereas in mean blood pressure no effect was found. Overall, physiological parameters showed that nature VR stimulation by using head-mounted displays revealed the greatest effect on the parasympathetic nervous system and thus was in line with the stress reduction theory suggestion that natural environments are the most restorative environment [15,16,27]. 

The effect on the parasympathetic nervous system may not result just from the natural environment, but may also be influenced by the head-mounted display and the noise-cancelling headphones, which helped to reduce the sensory overload (eg, alarms, noise) and deprivation (eg, missing daylight) in the ICU. One may argue that this effect should also be present in the urban environment, but there is a significant difference between the stimuli of these environments. While the sound of the natural environment mostly consisted of crashing waves and animal sounds, the soundscape of the urban scene was overloaded with typical city sounds [28]. These sounds may be as irrelevant and annoying as the noises in the ICU and therefore also cause sensory overload. Furthermore, due to memories and past experiences in the different environments, not all participants will respond the same to the different stimuli. Therefore, if in the past participants had a bad experience with certain stimuli inside a natural (eg, animals) or an urban environment (eg, car accident) it may evoke bad memories and thus influence physiological parameters negatively, with the opposite being true for positive experiences.

### Limitations

One of the limitations of this study is that all subjects were healthy subjects, so it is unclear whether the results could be transferred to critically ill patients. Another limitation was that cultural, living environment, and educational differences between the participants were recorded as a covariate but were not considered. Subjects from a rural origin may react differently to the stimulation and have different preferences than a subject who was born and grew up in a city. Furthermore, the different movement patterns inside the environments might have an influence on the perceived restorativeness.

### Conclusion

Overall, three environments that were different in technical setup and design were tested safely and successfully. We showed that VR stimulation by using a head-mounted display in combination with a dynamic, virtual, natural environment had the highest restorative effect on the physiological and psychological state of a subject. Furthermore, it was shown that the effect was not simply due to the isolation from the stressful environment but actually due to the composition of the natural environment. Therefore, the method of VR nature stimulation by using a head-mounted display may have potential as an early-intervention method directly used in the ICU to reduce sensory overload and deprivation and thus prevent neurocognitive late effects.

## Abbreviations

ANOVA: analysis of variance
Hz: hertz
ICU: intensive care unit
Inf: infinity
PRS-11: Perceived Restorativeness Questionnaire
SSQ: Simulator Sickness Questionnaire
VR: virtual reality
